# Model Organisms in Aging Research: Evolution of Database Annotation and Ortholog Discovery

**DOI:** 10.3390/genes16010008

**Published:** 2024-12-25

**Authors:** Elizaveta Sarygina, Anna Kliuchnikova, Svetlana Tarbeeva, Ekaterina Ilgisonis, Elena Ponomarenko

**Affiliations:** Institute of Biomedical Chemistry, 119121 Moscow, Russia; lizalesa@gmail.com (E.S.); a.kliuchnikova@gmail.com (A.K.); tarbeevasn@gmail.com (S.T.);

**Keywords:** aging, model organism, proteoforms, trend analysis

## Abstract

Background: This study aims to analyze the exploration degree of popular model organisms by utilizing annotations from the UniProtKB (Swiss-Prot) knowledge base. The research focuses on understanding the genomic and post-genomic data of various organisms, particularly in relation to aging as an integral model for studying the molecular mechanisms underlying pathological processes and physiological states. Methods: Having characterized the organisms by selected parameters (numbers of gene splice variants, post-translational modifications, etc.) using previously developed information models, we calculated proteome sizes: the number of possible proteoforms for each species. Our analysis also involved searching for orthologs of human aging genes within these model species. Results: Our findings indicate that genomic and post-genomic data for more primitive species, such as bacteria and fungi, are more comprehensively characterized compared to other organisms. This is attributed to their experimental accessibility and simplicity. Additionally, we discovered that the genomes of the most studied model organisms allow for a detailed analysis of the aging process, revealing a greater number of orthologous genes related to aging. Conclusions: The results highlight the importance of annotating the genomes of less-studied species to identify orthologs of marker genes associated with complex physiological processes, including aging. Species that potentially possess unique traits associated with longevity and resilience to age-related changes require comprehensive genomic studies.

## 1. Introduction

Aging is a complex biological process that affects body systems at all levels, from molecular to systemic, and involves cumulative damages that lead to functional changes and increase risks of disease and mortality [1,2]. One of the major gerontology issues is elucidation of the molecular mechanisms of aging and identification of key genes whose expression and sequence changes affect aging rate and lifespan [3]. The scientific question raised by George Martin in 2000 regarding the complexity of the aging process and the number of mechanisms involved remains unresolved [4].

Model organisms are indispensable tools to investigate aging-associated genes because they enable exploring aging mechanisms similar to those in humans in more controlled conditions and in a shorter time [5]. Model organisms, such as mouse (*Mus musculus*), nematode (*Caenorhabditis elegans*), fruit fly (*Drosophila melanogaster*), and yeast (*Saccharomyces cerevisiae*), play a key role in studying the genetics of aging. These simple model systems can independently replicate all aging phenotypes, making them the most popular model organisms in aging research, as evidenced by numerous studies [6,7]. A search for the terms “mouse” and “aging” in the PubMed database yields approximately 74,000 results, which is the highest number among all other species. These organisms display conserved lifespan-regulating mechanisms, such as the insulin-like signaling pathway and autophagy pathways [8]. However, the question remains: which model organisms are most similar to humans in the number of orthologs of aging-associated genes, and which of them have the greatest variety of proteoforms?

The development of technologies, such as high-throughput sequencing and mass spectrometry, has enabled systematic analysis of model organism proteomes, including alternative splicing, post-translational modifications, and single-amino acid polymorphisms [9,10]. These data provide the basis for detailed assessment of the functional capabilities of organisms and enable surveying the breadth of the proteoform spectrum, i.e., the variety of protein variants that can be encoded by a single gene. Information resources, such as UniProtKB, play a key role in annotation of these data and are an important tool for investigation of genetic variety at the proteome level [11].

Long-lived proteins, such as collagen and nuclear pore complex proteins, demonstrate how non-enzymatic modifications, e.g., glycosylation and cross-links, accumulate with age and induce structural and functional changes in tissues [2]. These modifications are enhanced by interactions with reactive oxygen species (ROS) and reduce the regenerative capacity of cells, decreasing the resistance of proteins to aging and increasing the risk of pathologies such as fibrosis.

The purpose of this study was to assess the overlap between human aging genes and genomes of 30 model organisms using data on conserved orthologs. Unlike previous studies [6], which focused on a limited number of species, our research encompasses a wide range of organisms, including not only mice, Drosophila, and nematodes, but also more rare species to identify potential avenues for applicability in research. Information for the search of orthologous genes was obtained from a previously published work [12], which serves as a systematic review of research on the topic of human aging. We performed comparative analysis of two samples: a full set of 2227 selected human aging genes and a subset of 272 genes associated with model organisms. Using this approach, we are able not only to identify key models that may be used in human aging research but also to assess the exploration degree of organisms in terms of their proteomic characteristics, such as rates of splicing and post-translational modifications, which are also related to evolutionary interspecies differences. Information regarding the annotation of the genome is essential for accurate modeling of the molecular processes occurring in the human body under both normal and pathological conditions.

## 2. Materials and Methods

Information used in this analysis was borrowed from two large biological databases: UniProt (release 2024_04) [13] and Ensembl (release 2024_05) [14]. These resources provide the most complete and up-to-date data on proteins, their functions, and associated genomes, which allows us to conduct a comparative analysis with allowance for genomic and proteomic omics and ultimately obtain an end-to-end idea of the exploration degree of organisms. In the analysis, information from Uniprot was used for reviewed proteins (that were verified by experts). In addition to experimentally confirmed entries, Ensembl genes included entries generated automatically using bioinformatics prediction algorithms. Taxonomic identifiers of organisms from the NCBI Taxonomy Database were used in the analysis.

The number of proteoforms was calculated for humans and several model organisms most popular in aging research. The list of species selected for analysis is given in Table 1. The list of model organisms included 29 species with completely sequenced genomes. We selected the most frequent organisms in publications according to the PubMed/MEDLINE biomedical library by analyzing MeSH terms associated with the publications using the ScanBious system [15] for the query “proteomics in aging”. The selected organisms included members of the kingdoms of bacteria, fungi, plants, and animals.

The approach used in this study is based on the assumption that the number of proteoforms (*Np*) can be calculated as *Np*(*H*) = *f* (*p*; *H*), where *p* is a set of parameters downloaded from molecular biological information resources, and *H* is an information model describing the relationships among *p* parameters. Presumably, *p* parameter values are not constant over time because the databases are constantly updated. The list of *p* parameters used to estimate the number of proteoforms is given below:

- *N*—Number of protein-coding genes in the genome;
- *ASg*—Number of alternatively spliced genes;
- *ASd*—Fraction of alternatively spliced genes in the genome (ASg/N);
- *AS*—Number of amino acid sequences formed by alternative splicing;
- *ASav*—Average number of splice variants per gene (AS/ASg);
- *SAPg*—Number of genes containing single nucleotide substitutions leading to single-amino acid polymorphisms (SAPs);
- *SAPd*—Fraction of genes containing single nucleotide substitutions leading to single-amino acid polymorphisms (SAPs) in the genome (SAPg/N);
- *SAP*—Number of amino acid sequences containing SAPs;
- *SAPav*—Average number of SAP-containing sequence variants per gene (SAP/SAPg);
- *PTMg*—Number of genes corresponding to proteins with post-translational modifications (PTMs);
- *PTMd*—Fraction of genes encoding PTM-containing proteins in the genome (PTMg to N);
- *PTM*—Number of amino acid sequences containing PTMs;
- *PTMav*—Average number of PTM-containing sequence variants per gene (PTM/PTMg);
- *Np* (*H*)—Number of proteoforms (proteome width), H—information model.

In the study, we considered several computational information models from previously published studies by Ponomarenko et al. [16] and Sarygina et al. [17], which reflect hypothetical variants of a combination of molecular events leading to the emergence of proteoforms (Formulas (1)–(3)):

*N*𝑝𝑠1 = 𝑁 × (1 + 𝐴𝑆𝑑 × 𝐴𝑆𝑎𝑣 + 𝑆𝐴𝑃𝑑 × 𝑆𝐴𝑃𝑎𝑣 + 𝑃𝑇𝑀𝑑 × 𝑃𝑇𝑀𝑎𝑣) (1)

𝑁𝑝𝑠2 = 𝑁𝑝𝑠1 + 𝐴𝑆 × (𝑆𝐴𝑃𝑎𝑣 + 𝑃𝑇𝑀𝑎𝑣)(2)

𝑁𝑝𝑠3 = 𝑁𝑝𝑠2 + 𝑁 × 𝑆𝐴𝑃𝑎𝑣 × 𝑃𝑇𝑀𝑎𝑣 + 𝐴𝑆 × 𝑆𝐴𝑃𝑎𝑣 × 𝑃𝑇𝑀𝑎𝑣(3)

The distinctive feature of model 1 is that it suggests that changes in the primary protein structure (SAP or PTM) arise only in conditionally canonical variants of amino acid sequences, in contrast to model 2 where SAP and PTM also arise in splice variants. Model 3 suggests the co-emergence of PTM and SAP in any amino acid sequences.

All the calculated parameters for humans and model organisms are presented in Table S1. The proteoform diversity values obtained using the proposed three different models are provided in Section 3 (Table 2). These data were used to assess the contribution of various molecular events to the proteoform spectrum variety and to draw conclusions about the exploration degree of the organism.

An OrthoFinder tool (v2.5.5) [18] was used to search for ortholog genes to evaluate the overlap between human aging genes and genomes of other model species. Canonical protein sequences of the gene in fasta format were downloaded from the UniProtKB (Swiss-Prot) database for each taxon ID. The list of aging-associated genes (*n* = 2227) was derived from our meta-analysis of proteomic studies of human blood plasma [12]. To compile the final list, we used the results of 17 publications performed using Olink technology [19,20,21], SOMAscan technology [22,23,24,25,26,27], mass spectrometry [28,29,30,31,32,33], and various proteomic techniques [34,35]. The final list of human aging-associated genes included all identifiers that were related to aging in the above-mentioned studies. A list of 272 genes was produced by integrating the general list with the data from Lehallier et al., 2020 [26], which were found to display the relationship between the presented aging genes and the model organisms. The lists of aging genes are presented in the Supplementary Materials (Table S2).

## 3. Results

**Figure 1:**
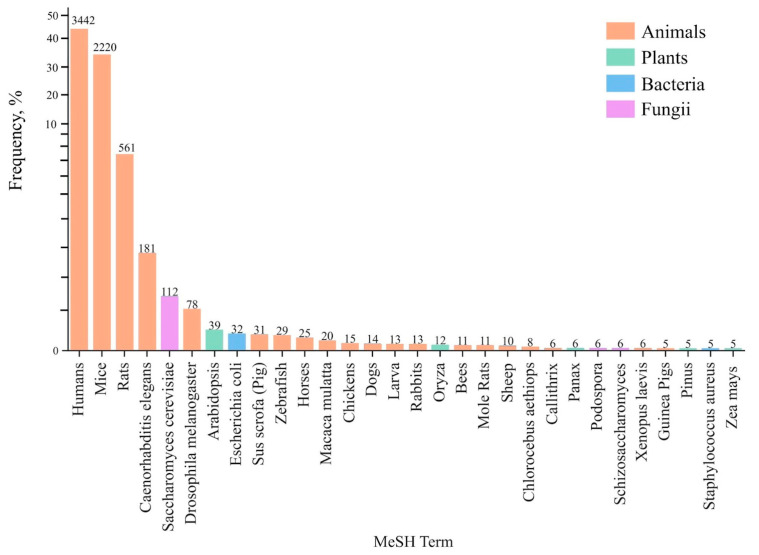
Top 30 selected MeSH terms associated with organisms that were accumulated by the query “proteomics in aging”. The x-axis shows MeSH terms, the y-axis shows frequency of occurrence, and the numbers above the columns show the number of publications.

To understand which model organisms are most often used in experiments on aging proteomics, we analyzed MeSH terms of publications in PubMed; its results are shown in Figure 1. For obvious reasons related to the significance of these studies for extending human lifespan, the MeSH term “Humans” comes to the fore. Studies involving humans enable direct investigation of age-related changes in the proteome and their association with diseases that progress over time, e.g., cancers, cardiovascular pathologies, and neurodegenerative disorders. The MeSH term “Mice”, as a separate model, appears in 2220 publications, which makes the mouse a key organism for experimental studies of aging; the C5BL inbred mouse strain stands out as one of the most commonly used strains due to its genetically homogeneous background.

It is worth noting the popularity of nematode *C. elegans* (181 publications) and yeast *S. cerevisiae* (101 publications) models and their important role in exploring the fundamental mechanisms of aging. These simple organisms enable investigating basic cellular processes at the molecular level. Their use complements research on more complex organisms, such as mice and rats. As can be seen from Figure 1, most of the selected MeSH terms have a more common or generic name of the model organism. Next, we selected the most popular species for each term, based on the list of the most representative species provided by Uniprot (https://www.uniprot.org/uniprotkb/statistics, accessed on 20 September 2024).

Further, we performed characterization of the 30 selected organisms, based on the exploration degree. The genome exploration degree of the model organisms was estimated proportionally to the number of genes in the genome, which had expert-verified annotations in the UniProtKB/SwissProt section (version 2024_04). The greater the number of expert-annotated genes in UniProtKB/SwissProt, the more studied the organism is. The fraction of annotated genes is the ratio of experimentally confirmed UniProtKB genes to all Ensembl genes (automatically generated gene annotations).

In more than 90% of organisms, the fraction of expert-annotated genes (entries in the UniProtKB/SwissProt section) is 10–20%. Only in 7 species (including humans and mice), the fraction of annotated genes in their genome is more than 75%. We compared data on the number of expert-verified annotations for genes of the most popular model organisms (see Table 1).

The data in Table 1 indicate that the genomes of more primitive species (e.g., bacteria or fungi) are characterized more completely than those of other organisms. If we exclude simple organisms from consideration, the only species with the completely annotated genome (about 100% of genes are expert-annotated and present in the UniProtKB/SwissProt section) is humans. Investigation of other species is, to some degree, aimed at gaining information about humans, their molecular functioning, and development of pathological conditions.

In the species 7–14 in Table 1, the genome is annotated in 10 to 90%. Expression products of the mouse genome are described most completely (82% of the genes have expert annotations in UniProtKB/SwissProt). Among plants, the most fully characterized genome is that of *A. thaliana* (>50% of genes are annotated). These also include the well-known model organisms, such as *D. melanogaster*, *C. elegans*, and *D. rerio*. The fraction of annotated genes in them is 27, 22, and 11%, respectively. The Zebrafish Genome Project was started 22 years ago [36]. Of the 30,000 protein-coding genes predicted in the *D. rerio* genome, 3300 genes have expert annotations in UniProtKB/SwissProt, despite the fact that about 70% of human genes have orthologs in the Zebrafish genome [36]. This species is most often used to model human genetic diseases, describing the relationship between mutations and phenotypic manifestations. Interest in studying molecular microheterogeneity at the protein level is only at the very beginning, as evidenced by a relatively small number of Zebrafish protein entries in proteomic databases.

Most of the organisms with less than 10% of annotated genes belong to the animal kingdom. In particular, these include the well-known model organisms, such as *S. scrofa* (Pig), *C. familiaris* (Dog), *E. caballus* (Horse), etc. However, these most informative models for human disease research are not in demand.

Data on the diversity of proteoforms (proteome width) of the selected model organisms are summarized in Table 2. The variety of the bacterial proteome is mainly related to the presence of SAPs and PTMs. Among fungi, *S. cerevisiae* and *S. pombe* are the most studied species. In the number of proteoforms encoded by one gene, *S. cerevisiae* is closest to animals: one *S. cerevisiae* gene is supposed to be capable of encoding 2 to 15 protein variants, which is comparable to that in *M. musculus* (see Table 2).

The largest calculated number of proteoforms is in mammals. In humans, this value ranges from 228,000 to 3,200,000 different forms, depending on the information model used (Table 2). The mouse genome, which contains about 18,000 genes, potentially encodes 750,000 different proteins. In plants, there are a slightly smaller number of proteoforms, about 400,000 (e.g., the genome of *A. thaliana* contains 16,000 coding genes). The lowest variety of encoded proteins is characteristic of organisms with a small number of protein-coding genes: yeast (*S. cerevisiae*) and bacteria, about 95,000 and 27,000, respectively. The estimated number of proteoforms in the naked mole-rat (*H. glaber*) (this organism has a minimal genome) is 16. According to UniProtKB/SwissProt data, there are only PTMs for proteins encoded by 4 out of 6 genes in this organism. Estimated parameters for all organisms can be found in the Supplementary Materials (Table S1).

In the context of human aging research, we propose a flexible approach to the selection of criteria for choosing model organisms, depending on the specific research task. Researchers may rely on several criteria: first, the simplicity of the organism; second, the degree of exploration of the organism, which may be assessed based on the coverage of available genomic and postgenomic data; third, the number of genes orthologous to human ones, which is important for cross-analysis of genes involved in common biological processes.

**Figure 2:**
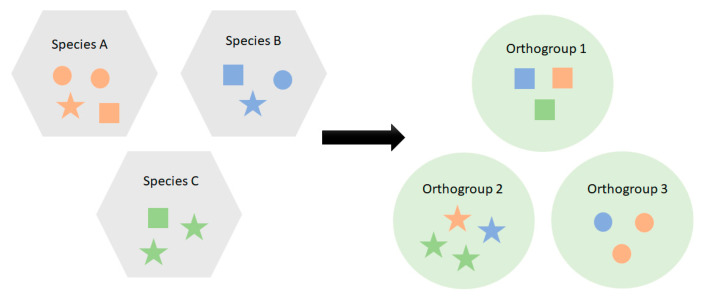
Schematic of orthogroup formation. Proteins from proteomes of different species are compared with each other in the format and are combined into orthogroups based on the similarity measure. Their number provides indirect judgment of the compared species proximity.

Evolutionarily related genes (homologs) in different species are often divided into pairs of genes that arose from speciation events (orthologs) and pairs of genes that arose from duplication events (paralogs) [37]. Orthologs tend to perform more similar functions than paralogs, as shown in studies [38,39,40]. A group of genes that descended from a common ancestor and retained their function in different species is called an orthogroup [18] (Figure 2).

The search for orthologs (orthogroups) was performed using the OrthoFinder tool [18], which enabled assessing the overlap between human aging genes and genomes of other model species (Table 3).

Table 3 presents the results of the search for orthologs for a group of human aging-associated genes using 30 model organisms. The study was conducted in two datasets: a full list of 2227 genes and a subset of 272 genes (see Section 2). The list of the identified ortholog genes is presented in the Supplementary Materials (Table S2).

The number of orthogroups, in which orthologs for human aging genes were identified, is presented for each model organism. For example, orthologs for 1827 and 1295 human aging genes were found in the mouse (*M. musculus*) and the rat (*R. norvegicus*), respectively, making them the top species for research in this field.

First of all, the high number of orthologs for aging genes in the above-described rodents is related to extensive studies of their genomes. The genomes of these species are annotated at a level close to that of humans, which enables reliable identification of orthologs and investigation of their function. This emphasizes their role as major model species for investigation of age-related diseases and processes.

Less studied and more distant evolutionary groups, such as insects and bacteria, display a lower degree of orthologous relationships with human aging genes, which limits their use in certain studies. In invertebrates, such as *C. elegans* and *D. melanogasterm*, the number of orthologs is lower than in mammals, despite the fact that they are widely used in aging research. This may be explained by both evolutionary differences and a less complete understanding of aging mechanisms in these groups. Plants (*A. thaliana* and *O. sativa*) and fungi (*S. cerevisiae* and *S. pombe*) demonstrate a limited number of orthologs for human aging genes, which is associated not only with the evolutionary distance but also with the features of their genomes. Although genomes of these organisms are relatively well studied, genetic conservation in terms of aging is low. However, they remain valuable for investigation of certain cellular mechanisms, such as stress responses and the cell cycle, which are also related to aging.

Organisms with less well-studied genomes, such as the honeybee (*A. mellifera*), as well as plant and invertebrate species, display a limited number of orthologs. This suggests that insufficient annotation of genomes and a limited understanding of their protein composition hinder full identification of orthologs for more complex processes, such as aging. Deeper annotation of these genomes might significantly contribute to aging research, especially if these species prove useful for specific aspects of aging.

## 4. Discussion

According to trend analysis, humans, mice, and rats are convenient and suitable for aging research. The opportunity to introduce DNA sequences of interest (e.g., genes) into the germline genome has made the mouse a powerful and indispensable experimental model in basic and medical research [41]. Introduction of human genes into the mouse genome to generate animal models that enable investigation of human-specific genes and diseases may be used to study aging-regulating signaling pathways or to test therapeutics [42].

Interestingly, the top 30 model organisms also include organisms more phylogenetically distant from humans, such as bacteria, fungi, and some plants. Recently, the rodent species *H. glaber* has increasingly been used to study the mechanisms of aging, demonstrating a phenotype of delayed aging and resistance to age-related functional diseases [43]. In this rodent, there are molecular mechanisms of resistance to chemical carcinogenesis induction via a weakened inflammatory response: loss of function in *RIPK3* and *MLKL* genes, which are required for necroptosis, reduces inflammation [44]. Also, a group of Csaba Kerepesi et al. [45] developed an epigenetic aging clock for *H. glaber*, which showed significant differences from mice and humans.

Technological progress and the emergence of omics technologies have led to accumulation of huge data. Understanding which organisms have been studied most deeply helps identify gaps in knowledge and, therefore, prioritize future research. We focused on the selected list of animal models and showed how the richness of genomic data directly affects possible size of the proteome.

Due to the dynamic nature that distinguishes the proteome from the genome, the proteome of biological objects is characterized by the diversity of proteoforms (different types of proteins) and the copy number of each proteoform. The capabilities of modern analytical techniques are not suitable for experimental assessment of the size of the proteome. To assess the diversity of proteoforms, we have proposed an information model and computing formulas that take into account the frequencies of mRNA splice variants, single-amino acid polymorphisms, and post-translational modifications, as well as the degree of organism exploration. The proposed approach provides theoretical assessment of protein diversity in the proteome of the organism under study. The analysis of UniprotKB annotations revealed that the number of interpretation options, i.e., proteoforms, not the number of protein-coding genes, plays a role in increasing the complexity of organisms. For example, the most studied bacteria and fungi shown in Table 1, due to a relatively small number of postgenomic events, have a smaller variety of proteoforms than animals (Table 2). We suggest the significance of the social component that reflects the degree of research interest and, thereby, the number of published research results and the fraction of annotated genes in the genome. This phenomenon was previously noted in a study involving scientific and technical big data [46].

The selection of model organisms strictly depends on the specific research objective. Having demonstrated the diversity and experimental exploration of proteomes of popular aging models, we characterized the species in terms of their genetic similarity to the human proteome, namely, to aging-associated protein-coding genes. Orthology analysis is based on the algorithms of alignment of model organism protein sequences with human aging proteins. Presumably, orthologous genes originating from a common ancestor play a similar functional role in different organisms. The absolute leaders are mice and rats because more than 75% of their genes are found in the orthologous groups of human aging genes, which makes these species the most suitable models for investigation of molecular processes. These models have a number of PCGs comparable to that in humans, and, unlike simpler models, such as Drosophila or nematode, have organ systems similar to human ones, both in function and in architecture. A fundamental tool to study the genetics of aging in mammals is identification of single-gene mutations that increase lifespan. A number of studies [47,48] have demonstrated relationships between individual genes (point mutation in the Prop1 gene, POU transcription factors) and lifespan processes in individuals. Genetically modified mice were also used to confirm previously discovered metabolic pathways involved in aging in Drosophila and *C. elegans* [49]. A search for orthologs also showed homology between humans and animals, such as *G. gallus*, *S. scrofa, X. laevis*, and *D. rerio*, as well as fungi *S. pombe* and *S. cerevisiae.* Yeast has played a crucial role in uncovering the molecular genetics of many basic cellular processes, such as the cell cycle [50], protein folding [51], intracellular trafficking [52], and many others. The genomic similarity (ortholog genes) between this very simple organism and mammals or even humans is surprisingly high, suggesting that this organism may be an effective model for human diseases, including aging. For example, two aging paradigms—replicative lifespan (RLS) and chronological lifespan (CLS) [53]—were discovered in yeast.

Our work helped reveal the orthology trend not only in popular models of aging but also in less trivial organisms, plants and bacteria. Species of these kingdoms display some similarity with human aging genes, which may potentially be used to consider them as experimental models. Simple model systems, despite their obvious morphological differences from humans, may be effectively used as a model to pave the way for future respective discoveries in humans.

## 5. Conclusions

The analysis of the trends in citations of model organisms in the literature identified the most common species that have attracted the attention of the scientific community in the context of aging research. In this study, we selected an integrative indicator of the level of organism research in the form of proteome size, expressed in terms of the number of alternative splicing events, post-translational modifications, and genomic variants. A large number of identified events implies significant scientific interest in the organism and is consequently associated with the number of experiments conducted, but does not reflect objective and complete biological events. We compared these proteomic characteristics with the number of orthologous genes associated with the development of age-related diseases, including aging, which we chose as an example of a multifaceted physiological process. This approach may also be applicable when planning research on model organisms in the context of different conditions or diseases.

Identification of aging ortholog genes confirmed the advisability of using certain model organisms as models to investigate lifespan. In addition to utilizing the most extensively studied models, it is crucial to emphasize the significance of annotating the genomes of less well-studied long-lived species. A comprehensive examination of these genomes may lead to the discovery of additional unique mechanisms specific to aging.

## Figures and Tables

**Table 1:** Number of genes annotated by UniProt database experts (presented in UniProtKB/SwissProt section) for the most popular model objects. PCG stands for number of protein-coding genes.

	Species	Taxon ID	Number of Genes (Ensembl)	PCG (UniProtKB/ SwissProt)	Percentage of Annotated Genes	Group
1	*Staphylococcus aureus*	1280	6220	10,245	164 *	Bacteria
2	*Escherichia coli* (strain K12)	83,333	5079	6066	119 *	Bacteria
3	*Homo Sapiens*	9606	19,846	20,429	103 *	Animal
4	*Saccharomyces cerevisiae*	559,292	6600	6727	101 *	Fungi
5	*Schizosaccharomyces pombe*	284,812	5145	5201	101 *	Fungi
6	*Pinus koraiensis* (Korean pine)	88,728	71	71	100	Plant
7	*Mus musculus* (Mouse)	10,090	21,700	17,228	82	Animal
8	*Arabidopsis thaliana* (Mouse-ear cress)	3702	27,655	16,389	59	Plant
9	*Rattus norvegicus* (Rat)	10,116	24,964	8205	32	Animal
10	*Drosophila melanogaster* (Fruit fly)	7227	13,986	3796	27	Animal
11	*Caenorhabditis elegans*	6239	19,985	4487	22	Animal
12	*Gallus gallus* (Chicken)	9031	17,077	2309	13	Animal
13	*Oryza sativa* subsp. *japonica* (Rice)	39,947	35,804	4190	11	Plant
14	*Danio rerio* (Zebrafish) *(Brachydanio rerio)*	7955	30,153	3343	11	Animal
15	*Sus scrofa* (Pig)	9823	22,040	1459	6.6	Animal
16	*Oryctolagus cuniculus* (Rabbit)	9986	20,599	978	4.7	Animal
17	*Canis lupus familiaris* (Dog)	9615	20,974	854	4	Animal
18	*Xenopus laevis* (African clawed frog)	8355	108,155	3507	3.2	Animal
19	*Panax ginseng* (Korean ginseng)	4054	6050	135	2.2	Plant
20	*Zea mays* (Maize)	4577	39,756	853	2.1	Plant
21	*Ovis aries* (Sheep)	9940	21,890	467	2.1	Animal
22	*Cavia porcellus* (Guinea pig)	10,141	18,095	306	1.6	Animal
23	*Macaca mulatta* (Rhesus macaque)	9544	21,591	365	1.6	Animal
24	*Equus caballus* (Horse)	9796	21,426	292	1.3	Animal
25	*Apis mellifera* (Honeybee)	7460	9935	99	1	Animal
26	*Podospora anserina*	515,849	10,544	100	0.9	Fungi
27	*Chlorocebus aethiops* (Green monkey)	9534	19,165	128	0.6	Animal
28	*Callithrix jacchus* (White-tufted-ear marmoset)	9483	21,965	110	0.5	Animal
29	*Triticum aestivum* (Wheat)	4565	145,065	381	0.2	Plant
30	*Heterocephalus glaber* (Naked mole-rat)	10,181	23,320	6	0.03	Animal

* Species annotated redundantly compared to Ensembl.

**Table 2:** Number of proteoforms (proteome width) of model organisms according to three computational information models (Formulas (1)–(3), Section 2). Both the total number of proteoforms for a model object and the average number of proteoforms encoded by one protein-coding gene (PCG) are given.

	Group	Species	PCG	Total Number of Proteoforms (per 1 PCG)		
				*N* *p* *s*_1_	*N* *p* *s*_2_	*N* *p* *s*_3_
1	Bacteria	*E* *. coli*	6066	7238 (1.2)	7368 (1.2)	37,528 (6.2)
2	Bacteria	*S* *. aureus*	10,245	11,254 (1.1)	11,254 (1.1)	59,712 (5.8)
3	Fungi	*P* *. anserina*	100	102 (1)	102 (1)	202 (2)
4	Fungi	*S* *. cerevisiae*	6727	14,794 (2.2)	16,572 (2.5)	100,972 (15)
5	Fungi	*S* *. pombe*	5201	7934 (1.5)	8188 (1.6)	37,196 (7.2)
6	Animal	*A* *. mellifera*	99	172 (1.7)	193 (1.9)	842 (8.5)
7	Animal	*C* *. elegans*	4484	7914 (1.8)	22490 (5)	69,332 (15.5)
8	Animal	*C* *. jacchus*	110	494 (4.5)	625 (5.7)	2072 (18.8)
9	Animal	*C* *. familiaris*	848	3510 (4.1)	4276 (5)	12,307 (14.5)
10	Animal	*C* *. porcellus*	306	931 (3)	1185 (3.9)	3272 (10.7)
11	Animal	*C* *. aethiops*	128	558 (4.4)	709 (5.5)	2578 (20.1)
12	Animal	*D* *. rerio*	3338	4483 (1.3)	7067 (2.1)	32,953 (9.9)
13	Animal	*D* *. melanogaster*	3796	12,844 (3.4)	35,464 (9.3)	151,871 (40)
14	Animal	*E* *. caballus*	292	1159 (4)	1302 (4,5)	5962 (20.4)
15	Animal	*G* *. gallus*	2309	4229 (1.8)	7058 (3.1)	33,259 (14.4)
16	Animal	*H* *. glaber*	6	13 (2.2)	13 (2.2)	13 (2.2)
17	Animal	*M* *. mulatta*	365	1374 (3.8)	1851 (5.1)	9108 (25)
18	Animal	*H* *. sapiens*	20,429	228,613 (11.2)	722,907 (35.4)	3,245,524 (158.9)
19	Animal	*M* *. musculus*	17,775	82,775 (4.7)	202,478 (11.4)	749,909 (42.2)
20	Animal	*O* *. cuniculus*	978	4381 (4.5)	5598 (5.7)	19,569 (20)
21	Animal	*O* *. aries*	467	1620 (3.5)	1927 (4.1)	8340 (17.9)
22	Animal	*R* *. norvegicus*	8202	38,059 (4.6)	60,773 (7.4)	217,124 (26.5)
23	Animal	*S* *. scrofa*	1459	6486 (4.4)	7600 (5.2)	27,664 (19)
24	Animal	*X* *. laevis*	3507	4814 (1.4)	6734 (1.9)	47,145 (13.4)
25	Plant	*A* *. thaliana*	16,431	29,015 (1.8)	76,736 (4.7)	395,227 (24.1)
26	Plant	*O* *. sativa*	4190	5256 (1.3)	7902 (1.9)	28,387 (6.8)
27	Plant	*P* *. ginseng*	135	150 (1.1)	159 (1.2)	159 (1.2)
28	Plant	*P* *. koraiensis*	71	82 (1.2)	82 (1.2)	82 (1.2)
39	Plant	*T* *. aestivum*	381	505 (1.3)	505 (1.3)	3267 (8.6)
30	Plant	*Z* *. mays*	853	1152 (1.4)	1560 (1.8)	12,859 (15.1)

**Table 3:** Comparison of aging gene group by model organisms.

Species	Number of Orthogroups in Which Human Aging Genes Are Present	
	Human Age-Associated Plasma Proteins (*n* = 2227)	Relevant Aging or Health Association in Model Organisms [26] (*n* = 272)
*M* *. musculus*	1827	253
*R* *. norvegicus*	1295	213
*G* *. gallus*	552	116
*C* *.s elegans*	496	102
*S* *. scrofa*	494	114
*D* *. melanogaster*	452	95
*X* *. laevis*	446	91
*D* *. rerio*	438	103
*S* *. pombe*	381	62
*A* *. thaliana*	377	68
*S* *. cerevisiae*	354	56
*O* *. cuniculus*	346	78
*C* *.familiaris*	310	79
*O* *. sativa*	185	36
*O* *. aries*	165	48
*E* *. caballus*	148	44
*M* *. mulatta*	144	38
*C* *. porcellus*	121	38
*E* *. coli*	115	19
*Z* *. mays*	85	16
*C* *. jacchus*	61	20
*C* *. aethiops*	58	24
*S* *. aureus*	58	12
*T* *. aestivum*	44	13
*A* *. mellifera*	17	8
*P* *. anserin*	16	3
*H* *. glaber*	5	0
*P* *. ginseng*	5	0
*P* *. koraiensis*	4	0

## Data Availability

The original contributions presented in this study are included in the article and Supplementary Material. Further inquiries can be directed to the corresponding author.

## Supplementary Materials

The following supporting information can be downloaded at: https://www.mdpi.com/article/10.3390/genes16010008/s1, Table S1. Estimated parameters for information models of the theoretical diversity of human proteoforms and model organisms. Table S2. Found orthogroups of human aging genes and model organisms.
